# Electronic Cigarette Trial and Use among Young Adults: Reasons for Trial and Cessation of Vaping

**DOI:** 10.3390/ijerph121215039

**Published:** 2015-12-17

**Authors:** Lois Biener, Eunyoung Song, Erin L. Sutfin, John Spangler, Mark Wolfson

**Affiliations:** 1 Center for Survey Research, University of Massachusetts Boston, Boston, MA 02125, USA; 2 Department of Social Sciences and Health Policy, Wake Forest School of Medicine, Medical Center Boulevard, Winston-Salem, NC 27157, USA; esong@wakehealth.edu (E.S.); esutfin@wakehealth.edu (E.L.S.); mwolfson@wakehealth.edu (M.W.); 3 Department of Family and Community Medicine, Wake Forest School of Medicine, Medical Center Boulevard, Winston-Salem, NC 27157, USA; jspangle@wakehealth.edu

**Keywords:** electronic cigarettes, surveillance, harm reduction

## Abstract

This paper identifies predictors of trial and current use, and reasons for trying and ceasing use of electronic cigarettes (e-cigarettes) among young adults, with particular attention to former and never smokers. Data are from a mail survey of a population-based sample of adults aged 18 to 35 (*N* = 4740) in three U.S. metropolitan areas. Survey items assessed trial and use of e-cigarettes, cigarette smoking status, and reasons for trial and for ceasing use of e-cigarettes. Almost 23% reported trial of e-cigarettes, and 8.4% reported using them in the past month. Current smokers were much more likely to have tried e-cigarettes (70.2%) than both former (32.3%) and never smokers (7.6%; *p* < 0.001) and to have used them in the past month (30.8%, 10.1%, 2.0% respectively; *p* < 0.001). Smoking status and scores on sensation seeking were significant independent predictors of both trial and current use of e-cigarettes. Never-smokers cite curiosity as the reason for trying e-cigarettes and also that their friends used them. The most frequent reason for ceasing use among never and former smokers was health concerns. For virtually none of them were e-cigarettes their first exposure to nicotine.

## 1. Introduction

Electronic cigarettes (“e-cigarettes”) are becoming increasingly popular, especially among younger adults, who have been shown to have the highest prevalence of vaping compared to all adults [1,2]. The high rate of use in this cohort is readily understood in the context of their high rate of cigarette smoking [3] and their attraction to other novel tobacco products, such as snus [4,5] dissolvable tobacco [6] and hookah [7].

Although long-term effects of e-cigarette usage are not known at this early stage of their existence, examinations of their content and biomarkers of toxicity among users [8,9] suggests that they are substantially less harmful than conventional cigarettes, that lung function improves significantly when smokers switch to e-cigarettes [10] and that for some, they may be effective aids to smoking cessation [11,12,13]. However, there are still concerns that their attractiveness to youth and young adults could result in net population harm if they serve as a pathway into nicotine addiction, which may eventually lead to use of cigarettes. Coleman *et al.,* 2015 [2] report that young adult non-smokers who try e-cigarettes are significantly more likely to demonstrate openness to future cigarette smoking. They report, in addition, that 86% of this group report having experimented with cigarettes in the past, making it difficult to establish the direction of any causal link between e-cigarette use and openness to smoking. Nevertheless, it is important to examine the predictors of use among young adults, and, in particular, to investigate their use among those who are not current smokers.

This paper explores prevalence of e-cigarette trial and use in population-based samples of adults, aged 18 to 35, in three metropolitan areas. It identifies predictors of e-cigarette trial and current use among smokers, former smokers, and never smokers, and compares their reported reasons for using and ceasing use of e-cigarettes.

## 2. Methods

### 2.1. Sample Design

Data for the study were from a population based mail survey of adults aged 18 to 35 residing in three metropolitan areas: Charlotte, NC, Denver, CO, and Topeka, KS. These areas were selected because they were all test markets for dissolvable tobacco products [14] which were the main focus of the survey. 

In order to balance the need for a representative sample and to keep costs down, we used a dual-frame sample, part random address-based sample (ABS) from the U.S. Postal Service Delivery Sequence File and part list frame. An ABS-only random sample would have a less complex sample design for obtaining generalizable estimates of the population, but because we are focusing on young adults, relying on a random ABS only would be inefficient and very expensive. Dual frame samples allow increased efficiency and decreased costs by supplementing the random sample with a list sample that has already been identified to include households likely to have a resident in the targeted age group. The list of addresses was purchased from Marketing Systems Group. It is based on a variety of publicly available and commercial sources, such as driver’s license registries, credit agencies, and marketing databases. Conducting a survey with a dual frame sample involves: (a) removing all addresses from the ABS sample that appear on the list sample; and then (b) drawing unduplicated samples from the three areas, Charlotte, Denver, and Topeka (approximately one-third the sample in each area) from the two sources—the ABS and the purchased list. The survey is conducted with respondents from each of the sources, and with the known probabilities of selection of each household in each frame, weights are developed so that the combined data can be used to make population-based estimates. Using this dual frame design, all residences within the designated geographic areas have a known probability of selection; no one residence excluded—even those with no landlines. By combining the two frames, we do not rely exclusively or too heavily on the cost-saving list sample and its unknown biases. Post-stratification weight adjustments (e.g., by gender, age, race and education) were developed to yield sample estimates that accurately reflect the population. The total sample included 31,999 households (4001 surveys were not delivered due to bad addresses.)

### 2.2. Survey Protocol

Data were collected between September and December 2013. There were up to five mailings per address: (1) An advance letter was sent to each household introducing the study and informing the recipient that only those residents who were aged between 18 and 35 were eligible to participate. A postcard, which could be used to declare that the household contained no age-eligible residents, was included in the mailing; (2) The first survey mailing included a cover letter and a one-dollar bill as a “token of appreciation.” The letter explained that when a completed survey was received, the household would be entered into a lottery with a 1 in 10 chance of winning a $50 gift card. The first question on the survey asked about the number of individuals in the household who were between 18 and 35. If the answer was “0”, the respondent was asked to stop there, and send back the blank questionnaire in the envelope provided. If more than 1, the person with the closest birthday was to fill out the questionnaire; (3) A thank you/reminder post card was mailed approximately two weeks later to all eligible households; (4) A second mailing of the survey was sent to non-respondents approximately one month later; (5) A post-card with a URL for online administration was sent to all non-respondents approximately 3 weeks after the second survey mailing. The study protocol was approved by the Wake Forest School of Medicine Institutional Review Board.

### 2.3. Measures

Tobacco Use Status: Respondents indicated any lifetime use of a list of 6 types of tobacco, as well as which was the first product tried (including e-cigarettes). Those who indicated any tobacco use were asked if they had smoked 100 cigarettes, and whether they currently smoked every day, some days, or not at all. Those responding affirmatively to the 100 cigarettes question, and checking either “every day” or “some days,” were defined as current smokers. Those who responded affirmatively to the 100 cigarette question, and indicated they now smoked “not at all,” were defined as former smokers. Those who denied having smoked 100 cigarettes in their lifetime, or who said that they had “never used any tobacco products,” were defined as never smokers. Three levels of smoking intensity were coded based on the average number of cigarettes smoked per day: High (16+); Medium (10 to 15); Low (<10). 

Expectations of Quitting Smoking*:* Current smokers were asked how likely they were to still be smoking in 12 months. Those responding “very” or “somewhat” likely were defined as unlikely to quit; those responding “Not very” or “not at all” were classified as likely to quit.

Electronic Cigarette Use: Those who indicated that they had used an e-cigarette “even one time” were defined as having tried an e-cigarette. Those who had tried were asked on how many of the previous 30 days they had used an e-cigarette. If they indicated use on 1 or more of the past 30 days, they were defined as current e-cigarette users. Three levels of frequency of e-cigarette use, based on number of days used in the past month, were computed: None (never tried or tried, but not used in the past 30 days), Medium (1 to 19 days), High (20 to 30 days).

Reasons for Trying E-cigarettes. Those who had tried e-cigarettes were presented with 7 possible reasons for trying and asked to check all that applied. The reasons included curiosity, use by friends, health risks relative to cigarettes, absence of smell, for use where smoking is banned, and to quit or cut down on smoking. 

Reasons for Stopping E-cigarette Use*.* Those who indicated that they had stopped using e-cigarettes were presented with 8 possible reasons for stopping, and asked to check the most important reason. The reasons included health concerns, negative reactions to taste and feeling sick, inferiority to other forms of tobacco, expense, lack of availability and social disapproval. 

Sensation Seeking. Sensation seeking was measured using the Brief Sensation Seeking Scale [15]. Using a five-point Likert scale (1 = strongly disagree to 5 = strongly agree), the eight-item scale measures agreement with statements such as: “I would like to explore new places” and “I prefer friends who are exciting and unpredictable”. Total sensation seeking scores were calculated as the average of all items for individuals who answered a minimum of five questions on the scale. The Cronbach’s alpha for this sample was 0.78.

Demographic Characteristics. The following demographics were measured: gender, age group (18–24), (25–30), (31–35), and race/ethnicity (white non-Hispanic *vs.* minority).

### 2.4. Analysis Plan

The data were weighted to account for the probability of selection and the sample design. Post-stratification weights were applied to ensure that the sample matched the population in terms of age, gender, race/ethnicity and education. Multivariable logistic regression was used to determine predictors of e-cigarette trial and current (*i.e*., past month use of e-cigarettes for the samples as a whole and for the subset of current smokers). Descriptive statistics (percentages and 95% confidence intervals for the percentages) were used to compare reasons for e-cigarette trial and for ceasing use of e-cigarettes. The Rao-Scott Chi Square test was used to determine the significance of differences in reasons endorsed by e-cigarette users of different smoking statuses when the cell sizes were 25 or higher.

## 3. Results and Discussion

The overall response rate was 34.9%. A total of 5105 respondents completed the survey, and we included 4740 who did not have any missing data for the main outcomes and covariables. The sample was composed of 18.3% current smokers, 14.5% former smokers, and 67.3% never smokers. 

Among the sample as a whole, 22.6% reported having ever tried e-cigarettes, and 8.4% reported using them in the past month. Among current e-cigarette users, the mean number of days used in the past month was 10, with 76% of current users reporting fewer than 20 days of use, and 24% reporting 20 or more days.

Current smokers were much more likely to have tried e-cigarettes (70.2%) than both former (32.3%) and never smokers (7.6%; *p* < 0.001) and to have used them in the past month (30.8%, 10.1%, 2.0% respectively; *p* < 0.001).

### 3.1. Predictors of E-Cigarette Trial and Current Use

Multivariate logistic regression demonstrated that in the sample as a whole, smoking status and sensation-seeking predicted both e-cigarette trial and past month use. Controlling for age, gender and race/ethnicity, smokers were more than 28 times as likely as never smokers to have tried e-cigarettes, and more than 19 times as likely to have used them in the past month; former smokers were about 6 times as likely to have tried e-cigarettes as never smokers, and about 5 times as likely to have used them in the past month. With respect to sensation seeking, for each point increase in the mean score, the individual was about one and a half times as likely to have tried e-cigs and to be a past month user. Age group was a significant predictor of trial (18 to 24 year olds more likely to try e-cigarettes than 31 to 35 year olds), but not current use. (See Table 1).

**Table 1:** Logistic regression analysis of electronic cigarette trial and current use among 4740 young adults.

Variable	E-Cigarette Trial		Past Month E-Cigarette Use	
	AOR	95% CI	AOR	95% CI
Cigarette smoking status				
Current smoker *vs.* Never smoker *	28.82 ^†^	20.13–41.26	19.68 ^†^	11.76–32.94
Former smoker *vs.* Never smoker *	6.36 ^†^	4.34–9.33	5.22 ^†^	2.78–9.77
Age				
Age 18–24 *vs.* Age 31–35 *	1.79 ^†^	1.23–2.60	1.13	0.71–1.79
Age 25–30 *vs.* Age 31–35 *	1.32	0.93–1.86	0.77	0.49–1.22
Gender				
Male *vs.* Female *	1.14	0.86–1.51	1.38	0.94–2.02
Race/Ethnicity				
Non-Hispanic White *vs.* Others *	0.90	0.62–1.29	0.94	0.59–1.50
Sensation seeking scale mean score	1.62 ^†^	1.32–1.97	1.49 ^†^	1.12–1.98

Notes: AOR = Adjusted Odds Ratio; 95% CI = 95 percent confidence interval; * reference group; ^†^ *p*-value < 0.05.

Examination of predictors among current smokers included the variables discussed above, as well as level of daily smoking and likelihood of quitting within 12 months. These analyses showed no significant predictors of e-cigarette trial or past month use. 

### 3.2. Reasons for Trying and Stopping Use of E-cigarettes by Smoking Status

As shown in Table 2, curiosity was the predominant reason endorsed for trying e-cigarettes, regardless of smoking status, but never smokers were significantly more likely to endorse this reason (77.3%) than former and current smokers (59% and 61% respectively). Among never smokers who had tried an e-cigarette, the next most frequently endorsed reason was that their friends use it (46%), followed by the lack of a noxious odor (25.9%). Among never smokers who had given up use of e-cigarettes, the two most frequently cited reasons were the concern that it was bad for health (47.8%) and that it tasted bad (15%). (See Table 3).

**Table 2:** Percent endorsing various reasons for trying electronic cigarettes by smoking status.

	Current Smoker (*N* = 531)		Former Smoker (*N* = 187)		Never Smoker (*N* = 199)		*p*-Value ^c^
	N ^a^	% ^b^ (95% CI)	N ^a^	% ^b^ (95% CI)	N ^a^	% ^b^ (95% CI)	
Curious	313	61.2 (54.6%–67.8%)	110	59.1 (47.9%–70.3%)	142	77.3 (68.5%–86.1%)	0.04
Better for health than cigs	312	55.1 (48.2%–61.9%)	74	42.7 (30.8%–54.5%)	26	17.2 (8.2%–26.3%)	<0.0001
Friends use it	145	30.9 (24.6%–37.3%)	58	28.1 (18.7%–37.5%)	94	46.0 (35.3%–56.7%)	0.02
Can use in no-smoking areas	229	43.1 (36.4%–49.8%)	43	33.8 (21.7%–46.0%)		NA	0.18 ^d^
Help to quit smoking	211	35.9 (29.7%–42.1%)	71	40.1 (28.5%–51.8%)		NA	0.53 ^d^
Cut down on smoking	219	41.1 (34.5%–47.6%)	35	18.5 (9.9%–27.0%)		NA	<0.0001 ^d^
Doesn’t smell bad	217	42.7 (36.0%–49.3%)	63	37.8 (25.9%–49.8%)	40	25.9 (15.7%–36.1%)	0.04

Notes: ^a^ Unweighted count; ^b^ Weighted percent; ^c^ *p*-value from Rao-Scott modified chi-square statistic; ^d^ *p*-value from Rao-Scott modified chi-square statistic among current and former smoker samples; NA = Not applicable.

**Table 3:** Most important reason for giving up electronic cigarette use by current smoking status.

	Current Smoker (*N* = 234)		Former Smoker (*N* = 114 )		Never Smoker (*N* = 131)	
	N ^a^	% ^b^ (95% CI)	N ^a^	% ^b^ (95% CI)	N ^a^	% ^b^ (95% CI)
Not strong enough	49	18.0 (10.1%–25.9%)	9	4.7 (0.8%–8.6%)	4	4.7 (0.0%–10.9%)
Bad for health	17	10.4 (4.1%–16.6%)	54	44.1 (29.3%–58.9%)	67	47.8 (37.8%–61.0%)
Didn’t like taste	22	7.7 (2.8%–12.5%)	11	9.5 (0.5%–18.4%)	14	15.1 (3.7%–26.5%)
Made feel sick	12	7.2 (1.8%–12.6%)	8	17.4 (1.7%–33.0%)	7	8.9 (0.0%–19.3%)
Couldn’t find in store	6	3.9 (0.0%–7.8%)	1	0.5 (0.0%–1.6%)	2	5.3 (0.0%–12.5%)
Like other tobacco better	61	23.9 (15.1%–32.7%)	16	14.1 (4.8%–23.3%)	7	3.6 (0.0%–7.7%)
Too expensive	65	28.8 (20.0%–38.1%)	12	6.6 (0.0%–13.1%)	16	9.1 (3.0%–15.7%)
Friends/family disapproved	2	0.1 (0.0%–0.4%)	3	3.2 (0.0%–7.8%)	14	5.4 (0.9%–10.0%)

Notes: Sample includes only those who tried e-cigarettes, and report having given up using them. Responses were mutually exclusive; 95% CI = 95 percent confidence interval; ^a^ Unweighted N; ^b^ Weighted percentage.

Among former smokers, after curiosity, the most frequently endorsed reasons for trying e-cigarettes was the belief that it was better for their health than regular cigarettes (42.7%) and that it might help them to quit smoking (40.1%). Among their reasons for giving up e-cigarettes, former smokers were most likely to endorse that it was bad for their health (44%), followed by the fact that it made them feel sick (17.4%) and that they preferred another form of tobacco (14.1%). 

Among current smokers, curiosity (61%), belief that it was better for their health than cigarettes (55%), its usefulness in no-smoking areas (43.1%), and lack of odor (43%) were the top four reasons endorsed. Among those current smokers who continued to smoke cigarettes, but gave up e-cigarettes, the top reasons were that it was too expensive (29%), they liked another form of tobacco better (24%) and it was not strong enough (18%).

### 3.3. First Product Tried

Among all who tried e-cigarettes, the most frequently reported initial product tried was cigarettes or cigars among smokers (93.1%), former smokers (92.4%) and never smokers (81.2%). Only 0.4% of never smokers (*n* = 4) reported that e-cigarettes was the first product tried.

### 3.4. Limitations

There are several important limitations in the current study which should be taken into account. First, it did not use a national sample; while it is representative of the three diverse metropolitan areas surveyed, it does not necessarily represent all young adults in the U.S. Second, the number of questions on e-cigarette use was restricted in this survey, and there were no questions on the timing of smoking cessation among former smokers who were e-cigarette users. As a consequence, we are unable to determine the rate at which former smokers quit by using e-cigarettes. Indeed, among former smokers, frequently mentioned reasons for trying e-cigarettes were the belief that they were better for their health than tobacco cigarettes, that e-cigarettes might help them with quitting, and that they could use them where smoking was not permitted. This does suggest that at the time of their initial trial, they had been smokers. Nevertheless, the study uses a representative sample from three large metropolitan areas, and allows for a closer look at vaping among young adult non-smokers. Although the rather low response rate does raise concerns about potential bias in the sample, the post-stratification weighting should reduce the impact of any bias due to disproportionate age, gender and ethnicity among respondents. 

## 4. Conclusions 

In this sample of young adults, we found that smokers are most likely to try and to use electronic cigarettes, which is consistent with the findings of a number of recent studies [12,16,17,18]. We found no significant demographic predictors of e-cigarette trial and use among smokers, nor any relationship with intensity of smoking or intensions to quit smoking. Thus, among smokers, the appeal of these products appears to cut across gender, race, age and ethnic groups.

We also see that about one third of former smokers and over 7% of never smokers report having tried e-cigarettes, and current use is 10% and 2% in these groups, respectively. Similar findings have led others to raise concerns that e-cigarette use is a threat to public health because it can lead to combustible tobacco use, presumably among those who otherwise would not have become smokers [2]. However, it is extremely rare for e-cigarette use to be the first exposure to nicotine in young adults. It was reported by fewer than 0.4% of the never smokers, and none of the current and former smokers. This may be a cohort effect, and e-cigarette use as the initial exposure to nicotine may become more common as younger cohorts, who are experimenting with vaping at higher and higher rates, mature [19]. Nevertheless, in this cohort, 99.6% of young adult never-smokers who tried e-cigarettes had previously used or experimented with another form of tobacco, suggesting that in these young adults tobacco is a gateway to e-cigarettes, rather than the reverse.

The fact that only 2% of never smokers reported using an e-cigarette in the past month suggests that it may have little appeal for this population. A recent article by Amato and colleagues recommends using a more stringent definition of current electronic cigarette use to distinguish between infrequent and more frequent users [20]. If we restrict current use to those reporting use on more than 5 times in the past month, the rate of use among never smokers declines to 0.4%, a negligible level. The majority of never-smokers who do try e-cigarettes report discontinuing their use either because they felt it was bad for their health or they didn’t like the taste. 

The inclusion of a measure of sensation-seeking as a predictor of e-cigarette trial and use is an important contribution of this research. Sensation-seeking is known to be associated with attraction to novel stimuli, and also is a trait that tends to decline as one ages [21]. The finding that in the sample as a whole, sensation-seeking is a significant predictor of e-cigarette use helps to understand why never smokers, who are the bulk of the sample, would try these novel products. The fact that curiosity was cited as a motive by over 77% of never smokers, significantly more than current and former smokers reinforces the notion that a desire to have a new experience is the basis of their attraction.

Another concern about e-cigarettes is that they may provoke relapse in former smokers. A recent national cross-sectional study examined e-cigarette use among former smokers who had been quit for various periods of time. Use of e-cigarettes was extremely rare among those who had been quit for 4 years or longer [16]. This tends to refute the notion that quitters will be drawn back to nicotine addiction through e-cigarette use. In the present study, among the most frequent reasons former smokers gave for trying e-cigarettes was the belief that they are better for their health than smoking and that they might help them quit. This suggests that they were smokers when they first tried the product. Unfortunately, the survey did not gather data on when smoking cessation had occurred, but at least some of the former smokers may have quit with the use of e-cigarettes. 

In summary this study, in conjunction with others that show similar results, should reduce concern about e-cigarette use among young adults who have never been smokers. Although reasons for trial given by former smokers suggest that a large proportion of them were smoking at the time, our findings for former smokers are more ambiguous. There is much research that is still needed in order to better understand both the potential benefits and harms of e-cigarette use by young adults. Prospective studies following young adults over time are required to see whether smokers who use e-cigarettes are more or less likely to quit and whether former smokers who use e-cigarettes are more or less likely to sustain smoking cessation than former smokers who do not use e-cigarettes. It is also important that future work include more detailed measures of history of intensity of e-cigarette use, and not rely simply on ever use and past month use. 

## References

[1] Agaku I.T., King B.A., Husten C.G., Bunnell R., Ambrose B.K., Hu S.S., Holder-Hayes E., Day H.R. Tobacco Product Use Among Adults—United States. http://www.cdc.gov/mmwr/preview/mmwrhtml/mm6325a3.htm.

[2] Coleman B.N., Apelberg B.J., Ambrose B.K., Green K.M., Choiniere C.J., Bunnell R., King B.A. (2015). Association between electronic cigarette use and openness to cigarette smoking among US young adults. Nicotine Tob. Res..

[3] U.S. Department of Health and Human Services (1986). The Health Consequences of Involuntary Smoking: A Report of the Surgeon General.

[4] Biener L., Bogen K. (2009). Receptivity to taboka and camel snus in a U.S. test market. Nicotine Tob. Res..

[5] Biener L., McCausland K., Curry L., Cullen J. (2011). Prevalence of trial of snus products among adult smokers. Am. J. Public Health.

[6] Southwell B.G., Kim A., MacMonegle A., Porter L. Differing Predictors of Snus, Electronic Cigarette, and Dissolvable Tobacco Use and Implications for Communication Intervention. https://cdc.confex.com/cdc/nphic11/webprogram/Paper26936.html.

[7] Maziak W., Taleb Z.B., Bahelah R., Islam F., Jaber R., Auf R., Salloum R.G. (2015). The global epidemiology of waterpipe smoking. Tob. Control.

[8] Farsalinos K.E. (2014). Safety evaluation and risk assessment of electronic cigarettes as tobacco cigarette substitutes: A systematic review. Ther. Adv. Drug Saf..

[9] Hecht S.S., Carmella S.G., Kotandeniya D., Pillsbury M.E., Chen M., Ransom B.M.S., Vogel R.I., Thompson E., Murphy S.E., Hatsukami D.K. (2014). Evaluation of toxicant and carcinogen metabolites in the urine of e-cigarette users versus cigarette smokers. Nicotine Tob. Res..

[10] Polosa R. (2015). Electronic cigarette use and harm reversal: Emerging evidence in the lung. BMC Med..

[11] Biener L., Hargraves J.L. (2014). A longitudinal study of electronic cigarette use in a population-based sample of adult smokers: Association with smoking cessation and motivation to quit. Nicotine Tob. Res..

[12] Brown J., West R., Beard E., Michieb S., Shahaba L., McNeill A. (2014). Prevalence and characteristics of e-cigarette users in Great Britain: Findings from a general population survey of smokers. Addict. Behav..

[13] Bullen C., Howe C., Laugesen M., McRobbie H., Parag V., Williman J., Walker N. (2013). Electronic cigarettes for smoking cessation: A randomised controlled trial. Lancet.

[14] Wolfson M., Pockey J.R., Reboussin B.A., Sutfina E.L., Egana K.L., Wagonera K.G., Spanglerd J.G. (2014). First-year college students’ interest in trying dissolvable tobacco products. Drug Alcohol Depend..

[15] Hoyle R.H., Stephenson M., Palmgreen P., Lorcha E.P., Donohewc R.L. (2002). Reliability and validity of a brief measure of sensation seeking. Pers. Individ. Dif..

[16] Delnevo C.D., Giovenco D.P., Steinberg M.B., Villanti A.C., Pearson J.L., Niaura R.S., Abrams D.B. (2015). Patterns of electronic cigarette use among adults in the United States. Nicotine Tob. Res..

[17] Dockrell M., Morrison R., Bauld L., McNeill A. (2013). E-Cigarettes: Prevalence and attitudes in Great Britain. Nicotine Tob. Res..

[18] Grana R., Benowitz N., Glantz S.A. (2014). E-Cigarettes: A scientific review. Circulation.

[19] Krishnan-Sarin S., Morean M.E., Camenga D.A., Cavallo D.A., Kong G. (2014). E-Cigarette use among high school and middle school adolescents in Conneticut. Nicotine Tob. Res..

[20] Amato M.S., Boyle R.G., Levy D.T. (2015). How to define e-cigarette prevalence? Finding clues in the use frequency distribution. Tob. Control.

[21] Roth M., Hammelstein P., Brahler E. (2007). Beyond a youthful behavior style—Age and sex differences in sensation seeking based on need theory. Pers. Individ. Dif..

